# Utilization of HIV testing and counselling services by women with disabilities during antenatal care in Uganda: analysis of 2016 demographic and health survey

**DOI:** 10.1186/s12889-021-12045-4

**Published:** 2021-11-02

**Authors:** Hussaini Zandam, Ilhom Akobirshoev, Allyala Nandakumar, Monika Mitra

**Affiliations:** The Lurie Institute for Disability Policy, The Heller School for Social Policy and Management, Brandeis University, 415 South Street, MS 035, Waltham, MA 02453 USA

**Keywords:** Antenatal care, Disability, Utilization, Disparity, HIV, Testing and counselling services, Uganda

## Abstract

**Background:**

HIV testing and counselling during antenatal care (ANC) is critical for eliminating mother-to-child transmission of HIV. We investigated disparity in utilization of HIV testing and counselling services (HTC) between women with and without disabilities in Uganda.

**Methods:**

We conducted a retrospective study using the nationally representative 2016 Uganda Demographic and Health Survey. The study sampled 10,073 women between age 15–49 who had a live birth in the last 5 years. We estimated unadjusted and adjusted odds ratio for receiving pre-test HIV counselling, obtaining an HIV test result, and post-test HIV counselling by disability status using logistic regressions.

**Results:**

We found that women with disabilities were less likely to receive pre-test HIV counselling (59.6 vs 52.4), obtain an HIV test result (68.2 vs 61.4), receive post-test HIV counselling (55.5 vs 51.6), and all HTC services (49.2 vs 43.5). From the regression analysis, women with disabilities were less likely to receive pre-test counselling [AOR = 0.83; CI = 0.74, 0.93] and obtain an HIV test result [AOR = 0.88; CI = 0.78, 0.99].

**Conclusions:**

Our findings revealed that women with disabilities are less likely to receive HTC service during ANC and highlighted the need for disability-inclusive HIV and reproductive health services. Government, non-governmental organizations, and other stakeholders should consider funding inclusive campaigns and identifying other mechanisms for disseminating health information and behavioral interventions to women with disabilities.

## Introduction

The United Nations 2020 Global AIDS Update reported a remarkable achievement in the fight against the HIV epidemic [1]. Since the UNAIDS 90–90–90 initiative, which aim to bring HIV testing and treatment to the vast majority of HIV-infected people by the end of 2020, over 80% of people living with HIV were aware of their status and about 67% of them were on antiretroviral therapy [2]. Also, new global infections declined by about 40% from 2.9 million in 1997 to 1.7 million in 2019, and new infections among children dropped by 52% from 310,000 in 2010 to 150,000 in 2019. However, about 61% of the new infections among children occurred in sub-Saharan Africa, presumably through mother-to-child transmission of HIV (MTCT) [1]. Early detection of HIV through testing before and during pregnancy and subsequent initiation of antiretroviral therapy is effective in preventing MTCT of HIV [3, 4]. Antenatal care (ANC) provides an opportunity for HIV screening and integration of routine maternal and child health (MCH) services with HIV services and thus critical for prevention of MTCT [5, 6].

While there are uneven gaps in prenatal HIV testing in sub-Saharan Africa, the Southern and Eastern African regions, home to the largest number of people living with HIV (20.6 million) [7], have witnessed progress in HIV screening and prevention of MTCT, ensuring that pregnant women are tested for HIV and those diagnosed are placed on treatment [8–10]. Despite this progress there is a lack of evidence on the utilization of these services by women with disabilities who have the highest risk of HIV infection among the disability community [11–13]. HIV programming in Southern and Eastern African regions rarely include the interrelationship between disability and HIV [14], and even when they do there is limited provision for treatment, care and support for people with disabilities [15].

Uganda recorded one of the highest rates of HIV testing during ANC of about (81.5%) [16], achieved mainly through scale up of provider-initiated HIV testing and counselling (HTC) [17]. Pre-test counselling included explaining the patient’s right to opt-out as well as options for care if they are HIV infected [18]. Post-test counselling included a didactic session on prevention options, disclosure and partner testing. Several studies have shown increased uptake of HCT and access to services following the introduction of provider-initiated testing and counselling during antenatal period [19, 20]. Despite the progress in HIV testing and counselling in Uganda, there is a lack of evidence on the utilization of HTC among pregnant women with disabilities. Previous research suggests several gaps in HIV/AIDS knowledge and access to HIV services among people with disabilities [21, 22]. A recent study found that multiple overlapping and compounding layers of assumptions, including misinformation and stigmatizing cultural beliefs, have contributed to impediments in accessing HIV information and services among women with disabilities [23].

Uganda has demonstrated its commitment to disability rights by ratifying the United Nations Convention on the Rights of Persons with Disabilities, and establishing a comprehensive body of legislation, policies, and socioeconomic and health programs [24]. Specifically, Uganda’s HIV Counselling and Testing Policy explicitly recognizes and targets people with disabilities as a HIV high risk group for HTC services [25]. However, there is still a gap between laws, policies, and practice [24, 26]. The implementation gap is characterized by negative cultural attitudes toward disability, insufficient funding, insufficient training in inclusive education, and limited access to accessible information and assistive mobility devices, which are also associated with challenges in accessing reproductive health services among women with disabilities, further compounding potential disparities in HTC utilization during the antenatal period among women with disabilities [27, 28]. However, it is unclear how consistent HIV testing and counselling services are provided to pregnant women with disabilities.

The aim of this study is to investigate disparity in utilization of HTC services between women with and without disabilities in Uganda. We hypothesized that women with disabilities will have lower utilization of HTC services compared to women without disabilities.

## Materials and methods

### Data

We used cross-sectional data from the 2016 Uganda Demographic and Health Surveys (UDHS) [29]. The UDHS provides up-to-date estimates of key demographic, socioeconomic, and health indicators in Uganda, including sexual and reproductive health in adults, infant and maternal mortality, child mortality, nutritional status, malaria, and disability status. Detailed information about survey design are available in the UDHS final survey reports [29]. We conducted a retrospective analysis of the data to estimate the utilization of HIV testing and counselling services among women who had a live birth in the last 5 years prior to the survey.

### Sample

The UDHS data are nationally representative of Ugandan women 15–49 years of age. A total of 18,506 women participated and responded to the disability module in 2016. For this study, we excluded women who did not give birth in the last five years before the survey. Women who did not have any antenatal visit during their last pregnancy within five years (*n* = 4511) were also excluded. Our final analytic sample included 10,073 women who had at least one antenatal visit during their last pregnancy within 5 years preceding the survey, including 8470 women without disability and 1603 women with disabilities.

### Outcomes

The study outcome variables were components of the HTC during antenatal period and included the following: (1) receiving pre-test HIV counselling, (2) taking an HIV test and obtaining the result, and (3) receiving post-test HIV counselling. These outcomes were measured as binary outcomes (yes/no) where yes indicates a positive response whether women received pre-test HIV counselling, took a HIV test and obtained result, and received post-test HIV counselling. Additionally, we created a composite binary variable (yes/no), where yes indicates that women received all of the HTC components mentioned above.

### Disability

The primary explanatory variable was disability status and was assessed from responses to the Washington Group Short Set of Questions on Disability (WGSS) [30]. The WGSS is the standard approach to measuring disability in censuses and large surveys producing valid data that is internationally comparable [31]. Disability status assessment was based on experience of difficulties by an individual related to six functional areas including: (1) seeing; (2) hearing; (3) walking; (4) remembering; (5); communicating and (6) washing or taking care of self. Possible responses to the questions were as follows: no difficultly; some difficulty; a lot of difficulty; and cannot do at all. We employed the least restrictive recommendation from the Washington Group on Disability Statistics’ analytical guidelines in creating a dichotomous disability categorization [32]. Women who reported “some difficulty” or “a lot of difficulty” or “cannot function at all” to any of the six functional domains were classified as having a disability; women who reported “none” were classified as not having a disability [32].

### Covariates

We included the following sociodemographic characteristics as covariates in all our multivariate analyses: age (< 25 years, 25–34 years, 35+ years), education (no education, primary, secondary, higher), marital status (never married, formerly married/widowed, married/partnered), number of currently living children (0, 1, 2, 3, 4 or more), and employment status (i.e., employed or unemployed). Household characteristics included household wealth quintile (lowest, second, third, fourth, highest), and place of residence (i.e., urban or rural).

### Statistical methods

We compared sociodemographic and housing characteristics of women with and without disabilities using the chi-square test for categorical variables and a test for continuous variables. We also calculated the proportions for each outcome indicator between women with and without disabilities. Logistic regression models were used to estimate the unadjusted and adjusted odds ratios (with 95% confidence intervals) for each outcome using women without a disability as the reference group. We used Stata version 16 for all analyses, applying *svy* commands to account for the complex sampling design of the UDHS. *P*-value < 0.05 was used as the highest level of acceptable significance.

## Results

Table 1 presents descriptive statistics of the sample providing bivariate contrasts in sociodemographic and household characteristics between Ugandan women 15–49 years of age who were pregnant in the last 5 years by disability status. In comparison to women without disabilities, women with disabilities were more likely to be older, less educated, and less likely to be married. They were also more likely to have more children, be employed or working, live in poor households, and live in rural areas.

**Table 1:** Description of the Sample of Women 15–49 Years Old Who Were Pregnant in the Last 5 Years by Disability Status, Uganda, 2016

Characteristics	Disability status		***P***-value
	No ***n*** = 8470	Yes ***n*** = 1603	
**Women’s characteristics**			
Age			**< 0.000**
< 25	8.7	4.3	
25–34	71.8	62.3	
35+	19.4	33.4	
Age, Mean (SD)	29.7 (8.19)	33.8 (8.62)	**< 0.000**
Highest educational level			**< 0.000**
No education	9.6	15.5	
Primary	58.5	68.7	
Secondary	24.2	12.6	
Higher	7.7	3.2	
Current marital status			**0.004**
Never married	6.1	3.7	
Married	81.2	82.3	
Formerly married	12.7	14.0	
Number of currently living children			**< 0.000**
0	0.9	0.8	
1	22.8	11.8	
2	19.8	15.4	
3	17.0	14.3	
4	12.5	14.8	
5+	27.0	43.2	
Currently working			**< 0.000**
No	22.2	14.5	
Yes	77.8	85.5	
**Household characteristics**			
Wealth index			**< 0.000**
Lowest	20.5	22.7	
Second	19.4	26.5	
Middle	18.3	22.7	
Fourth	18.6	16.7	
Highest	23.2	11.2	
Residence			**< 0.000**
Urban	24.3	16.5	
Rural	75.7	83.5	

Source: Uganda Demographic and Health Survey (UDHS) 2016. *P*-values for differences, Chi2-test. Boldface indicates statistical significance (*p* < 0.05). *N* = 10,073 (Weighted Percentages)

Table 2 reports proportions, unadjusted and adjusted odd ratios, and respective 95% confidence intervals for HTC utilization by disability status. Compared to women without disabilities, fewer women with disabilities received pre-test HIV counselling, received an HIV test and obtained HIV test result, and received post-test HIV counselling. Similarly, compared to women without disabilities fewer women with disabilities received all of the HTC services.

**Table 2:** Proportions, Unadjusted and Adjusted Odd Ratios (with 95% Confidence Intervals). For HTC by Disability Status (*N* = 10,073)

HTC Indicator	No Disability (***n*** = 8470		With disability (***n*** = 1603)	
**Pre-test HIV counselling**				
Proportion^a^, 95% CIs	**59.6**	**58.2–61.1**	52.4***	**49.6–55.2**
uOR, 95% CIs	Referent		0.75***	**0.68–0.84**
aOR^b^, 95% CIs	Referent		0.83**	**0.74–0.93**
**HIV test and obtaining results**				
Proportion ^a^, 95% CIs	**68.2**	**66.9–69.4**	61.4***	**58.7–64.1**
uOR, 95% CIs	Referent		0.77***	**0.69–0.86**
aOR^b^, 95% CIs	Referent		0.88**	**0.78–0.99**
**Post-test HIV counselling**^**c**^				
Proportion ^a^, 95% CIs	**55.5**	**54.1–56.9**	51.6***	**48.4–54.4**
uOR, 95% CIs	Referent		0.85***	**0.76–0.94**
aOR^b^, 95% CIs	Referent		0.93	0.84–1.04
**All HTC services**				
Proportion ^a^, 95% CIs	**49.2**	**47.6–50.2**	43.5***	**40.6–46.4**
uOR, 95% CIs	Referent		0.80***	**0.72–0.89**
aOR^b^, 95% CIs	Referent		0.87***	**0.78–0.97**

Source: Uganda Demographic and Health Survey (UDHS) 2016. *** *p* < 0.01, ** *p* < 0.05, * *p* < 0.1

Notes: ^a^ Weighted proportions; ^b^Adjusted for maternal age, education, marital status, number of living children, employment, household wealth, and residence. Boldface indicates statistical significance (*p* < 0.05). ^c^Sample size (*N* = 6956). Abbreviations: HTC = HIV testing and counselling, uOR = unadjusted Odd Ratios, aOR = adjusted Odd Ratios, CIs = Confidence Intervals

Unadjusted analyses showed that compared to women without disabilities, women with disabilities had about 25% lower odds of receiving pre-test HIV counselling (OR = 0.75, 95% CI: 0.68–0.84 *p* < 0.001), 23% lower odds for receiving an HIV test itself and obtaining the HIV test result (OR = 0.77, 95% CI: 0.69–0.86 *p* < 0.001), 15% lower odds of receiving post-test HIV counselling (OR = 0.85, 95% CI: 0.76–0.94 *p* < 0.001), and had 20% lower odds of receiving all of the HTC services (OR = 0.80, 95% CI: 0.72–0.89 *p* < 0.001). Except for post-test HIV counselling, these differences remained robust and the lower odds only slightly attenuated after adjusting for sociodemographic and household characteristics.

## Discussion

In this secondary analysis of 2016 UDHS data, we found empirical evidence for significant differences in utilization of HTC services between disabled and non-disabled pregnant women in Uganda. Disabled women compared to their non-disabled peers were likely to be older, have lower level education, and come from poorer households. They were, however more likely to be employed, which reflects some level of empowerment. Our principal finding shows that disabled women were less likely to receive pre-test HIV counselling, to be tested for HIV, and receive post-test HIV counselling.

Uganda has been one of the few countries in sub-Sahara Africa to promote disability-inclusive HIV prevention services. For example, Uganda’s HIV Counselling and Testing Policy [25] explicitly recognizes that people with disabilities are at higher risk of HIV infection, and may experience difficulty in accessing HIV services. Further, this policy document stipulates that all HIV counselling and testing services should address the unique needs of persons with disabilities to address the persistent inequities [25]. Hanass-Hancock and colleagues [14] who studied eighteen national strategic plans for the inclusion of people with disabilities in HIV/AIDS services in Eastern and Southern Africa, found that Uganda’s national strategic plan represented the region’s best practice in disability-inclusive response to HIV/AIDS. Disability advocacy and self-advocacy groups and organizations in Uganda have been very active and engaged in developing disability-inclusive national strategic plans to respond to the HIV/AIDS epidemic [33].

Despite these efforts, our study found significant differences in utilization of HTC services between disabled and non-disabled pregnant women in Uganda. Disabled women compared to their non-disabled peers were less likely to receive pre-test HIV counselling, to be tested and received HIV result, and receive post-test HIV counselling. Our findings of persistent disability-related disparities in antenatal HTC service utilization points to a gap between disability-inclusive policy development and implementation in Uganda. While the laws and policies in several African countries, including Uganda, explicitly protect disability rights, equality, and nondiscrimination in access HIV services including testing and treatment [14], a study has documented several challenges and clear gaps in implementation of disability-inclusive HIV services, resulting in systemic inequities in HIV service utilization and care [34].

These persistent inequities in access to and utilization of HIV services among people with disabilities, among other things are rooted in assumption, misinformation and stigmatization about disability [23]. Research reveals a tendency of misconceptions about exposure to risk of HIV among people with disabilities [21], and also that the fear of stigma prevents people with disabilities from accessing HIV services, even when they consider that they may be HIV positive [35], This issue is compounded by the lack of disability awareness and training among health care providers, lack of accessible HIV and testing information and services, and communication barriers such as sign interpreters, making it difficult for people with disabilities to access and utilize HIV services [22, 33]. Widespread misconceptions about lower HIV exposure among women with disabilities allegedly for being non-sexual and dismissing evidence on higher exposure to sexual violence often lead to exclusion from health promotion activities, including HIV campaigns, which make women with disabilities disproportionately vulnerable to HIV, particularly young women with disabilities [36].

To ensure access to HIV health services for individuals with disabilities in Uganda, there is a need to increase awareness among reproductive-age women with disabilities about the risk of HIV and the importance of seeking HIV services. Equally important is to provide continuous training for healthcare providers about the risks of HIV among people with disabilities by providing tools to meet the unique needs people with disabilities to have equitable access to sexual and reproductive health. For example, a study found that providing disability-related training among health care providers can be effective not only in sensitizing health workers to improve access to HIV services for people with disabilities, but also in providing knowledge and skills to implement needed changes [37]. However, for a sustainable change, such endeavors require political will on the part of national and local governments and commensurate budget allocations.

### Limitations

The strength of the study lies in the use of nationally representative survey data and the large sample size. Notwithstanding, the study is without limitations. First, the UDHS does not include questions on the severity, duration, onset, and cause of disability—all of which may limit the sensitivity and accuracy of the data presented. Second, the current set of domains in the Washington Group questions and how they are administered may be inadequate for identifying the majority people with disabilities including those living in rural areas, refugee camps and those living in institutions. Third, the use of the cross-sectional study design, as employed in the DHS, a cause-and-effect relationship could not be determined. Fourth, the study adopted self-reporting of past events (e.g., prenatal care uptake or HIV testing), which is subjected to social desirability and recall bias. Lastly, there are several other unmeasured confounders such as couple testing, availability of testing services and how testing is done, which could potentially influence the uptake of HIV testing during the ANC visits.

## Conclusion

Our findings revealed disparities driven by disability status indicating that women with disabilities are less likely to receive HTC service during ANC. There’s a need for future research to understand from both women with disabilities and healthcare providers perspectives why pregnant women with disabilities were not receiving these services. Such findings can help providers plan an inclusive HIV and reproductive health services that could improve the uptake of HTC services among pregnant women with disabilities. Government and all other stakeholders should consider reforming health systems that can accommodate the needs of people with disabilities. This is necessary to guarantee healthcare for all in light of the COVID-19 Pandemic that disproportionately impacted healthcare access for people with disabilities.

## Data Availability

The datasets generated during and/or analyzed during the current study are available in the Demographic and Health Survey program website, https://dhsprogram.com/publications/publication-FR333-DHS-Final-Reports.cfm

## Abbreviations

AIDS: Acquired immune deficiency syndrome
ANC: Antenatal care
AOR: Adjusted odds ratio
CI: Confidence interval
HIV: Human immunodeficiency virus
HTC: HIV testing and counselling services
MCH: maternal and child health
MTCT: mother-to-child transmission
OR: Odds ratio
UDHS: Uganda Demographic and Health Surveys
UOR: Unadjusted Odd Ratios
WGSS: Washington Group Short Set
